# Classical heart rate variability and nonlinear heart rate analysis in mice under Na-pentobarbital and ketamine/xylazine anesthesia

**DOI:** 10.55730/1300-0144.5383

**Published:** 2022-02-05

**Authors:** Hasan KAZDAĞLI, H. Fehmi ÖZEL, Mustafa ÖZBEK, Şüheda ALPAY, Mürüvvet ALENBEY

**Affiliations:** 1 Vocational School of Health Sciences, İzmir University of Economics, İzmir, Turkey; 2 Vocational School of Health Sciences, Manisa Celal Bayar University, Manisa, Turkey; 3 Department of Physiology, Faculty of Medicine, Manisa Celal Bayar University, Manisa, Turkey

**Keywords:** Mice, anesthetics, heart rate variability, autonomic nervous system

## Abstract

**Background/aim:**

Anesthetics are often used in animal experiments to achieve immobilization and relieve pain. However, many anesthetics can alter the dynamics of cardiovascular systems. We aimed to compare the effects of two frequently used anesthetics agents on heart rate variability (HRV) parameters in mice.

**Materials and methods:**

This observational study was performed between May and June 2014 in 21 male BALB/c mice aged 16–20 weeks. The animals were divided into three groups: pentobarbital (P), (n = 7); pentobarbital+fentanyl (P+F), (n = 7); and ketamine+xylazine (K+X), (n = 7). Surface electrocardiography (ECG) electrodes were placed in lead II configuration. The tachogram of RR intervals was obtained after R waves were detected using the Pan-Tompkins real-time QRS recognition algorithm. Frequency-domain, time-domain, and nonlinear HRV analyses were performed.

**Results:**

The bradycardia effect was higher in the K+X group (p < 0.01). Time-domain indices were higher in group K+X compared to group P (p < 0.01) and group P+F (p < 0.001). Very low frequency (VLF) power was significantly lower in group K+X compared to group P and group P+F (p < 0.01). Low frequency (LF) power, low frequency/high frequency (LF/HF) ratio, and total power (TP) were higher in group K+X compared to group P (p < 0.01) and group P+F (p < 0.001). The detrended fluctuation analysis short-term parameter (DFAα_1_) was significantly higher in group K+X compared to group P+F (p < 0.05) and the long-term parameter (DFAα_2_) was lower in group K+X compared to group P (p < 0.05). Standard deviations SD1 and SD2 were higher in group K+X compared to group P (p < 0.001) and group P+F (p < 0.001), SD2/SD1 ratio was lower in group K+X compared to group P (p < 0.05) and group P+F (p < 0.05). Entropy measures did not differ between groups.

**Conclusion:**

HRV analyses, including nonlinear methods, indicated that a K+X combination reduces imbalance and disorder in the regulation of the autonomic nervous system (ANS) in comparison to both P and the P+F combination.

## 1. Introduction

The number of heart beats per minute is referred to as heart rate. The difference in the time intervals between adjacent heartbeats is defined as heart rate variability (HRV) [1]. Heart-brain interactions and complex nonlinear autonomic nervous system (ANS) processes produce HRV, which may be considered as a measure of neurocardiac activity. It is well known that the ANS persistently regulates and monitors almost every function of the body, and ANS disturbances occurring when the body is under stress or in a pathological state may alter HRV [2].

Animal studies have great importance for a better understanding of disease mechanisms and for the development of new therapeutic methods. Anesthesia and/or analgesia are often used in animal experiments to achieve sufficient immobilization, and relieve stress or pain [3]. However, any given anesthetic may change the dynamics of the physiological system [4], which may, in turn, falsify the results of the study. It has been reported that anesthetics may cause vagal withdrawal, which may be inappropriate for the studies that investigate the various aspects of ANS [5, 6]. Monitoring ANS via HRV analysis may be beneficial since this method is a relatively variable tool for evaluating ANS functions [7]. Therefore, in the studies monitoring ANS regulation via analysis of HRV, it is important to be aware of the effects of commonly used anesthetics.

Three anesthetics, pentobarbital and ketamine/xylazine, are commonly used in animal studies because of their favorable recovery profile and the relatively lower incidence of side effects [8]. However, induction of anesthesia with pentobarbital is often associated with a significant decrease in mean arterial blood pressure (BP), body temperature (BT), and heart rate (HR) [9]. The hypotensive effect of pentobarbital may be attributed to a decrease in systemic vascular resistance or cardiac output, altered baroreflex mechanisms, and decreased myocardial contractility [10,11]. Inhibition of the sympathetic nervous system may explain the pentobarbital-induced hemodynamic alterations, however, the exact mechanism is still unknown [12]. These effects of pentobarbital, doses of which range from 30 to 100 mg/kg in studies, are reported to be dose-dependent [11]. While the therapeutic dose of 30 mg/kg pentobarbital is reportedly free of depressive affect, it is considered that larger doses cause antiadrenergic effects on the cardiac system [5].

The sedative analgesic most used by veterinarians is xylazine, an alpha2-adrenoceptor agonist. Side effects include bradycardia, depression of the cardiopulmonary system, and cardiac dysrhythmia; xylazine works well with ketamine to provide muscle relaxation and analgesic effects [13].

Ketamine has several pharmacological characteristics, including its analgesic effects and cardiovascular stimulating effects, such as increasing BP, HR, and cardiac output at the normal doses [14]. Ketamine has a short anesthetic duration and muscular rigidity effect, for longer surgery durations, and its use alone may be ineffective; therefore, a combination of ketamine plus xylazine (K+X) is more appropriate. Also, this combination allows the usage of reduced doses, so the unwanted effects of both agents can be avoided [15].

Fentanyl citrate (F) is an effective, short-acting narcotic analgesic; however, it has been reported to trigger extended and recurring respiratory depression [16]. The accumulation of fentanyl in plasma and the central nervous system following a single intravenous bolus injection has been related to persistent ventilatory depression in anesthetized dogs [17,18].

This study was performed to compare the possible effects of commonly used anesthetic agents on HRV parameters in mice by conducting both classical frequency and time domain analyses, as well as nonlinear analysis.

## 2. Material and methods

### 2.1. Animals and anesthesia

The animal experiment of the present observational study was performed after obtaining the permission of the local Ethics Committee for Animal Experimentations (No:77.637.435-13) between May and June 2014. 16–20 weeks-old male BALB/c mice weighing 26.19 ± 2.11 grams were purchased from the KOBAY Incorporated Company (Ankara, Turkey). Since it has been reported that the menstrual cycle affects HRV analysis, males were selected [19].

The animals were caged for at least five days before initiation of experiments in a special room in the animal care center of Manisa Celal Bayar University (DEHAM-Manisa/Turkey) at a room temperature of 20–22 °C under 12-h light/dark cycles. Drinking water and food were provided ad libitum. All experiments were performed at the same time under daylight.

Before the experiments, each mouse was weighed to calculate the anesthetic/analgesic doses. The mice were anesthetized with an intraperitoneal injection of three different anesthetics. Anesthetic doses were selected as follows [20]: Na-pentobarbital (90 mgkg^−1^, i.p., SIGMA Inc. Germany) for group P; Na-pentobarbital (90 mgkg^−1^, i.p.) plus fentanyl (0.2 mgkg^−1^, i.p., GENESIS Inc. İstanbul-Turkey) for group P+F; and, finally, ketamine (100 mgkg^−1^, i.p., SIGMA Inc. Germany) plus xylazine (8 mgkg^−1^, i.p., SIGMA Inc. Germany) for group K+X. To assess the depth of anesthesia, both pedal withdrawal reflex and the breathing frequency were evaluated. Animals were placed on a heated thermal plate to avoid hypothermia due to the anesthesia. During experiments, the rectal temperature of the animals was observed and maintained around 36.5 °C.

### 2.2. Experimental design

**Figure 1:**
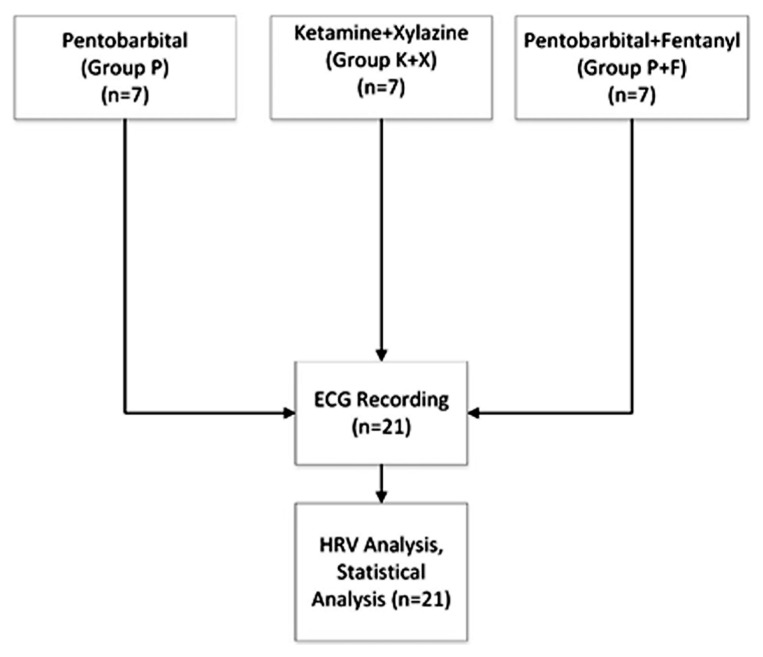
Study design.

To evaluate the effects of different anesthetics and analgesics on the heart rate variability and complexity in BALB/c mice, we constructed three groups as illustrated in Figure 1: A total of 21 mice were divided into the three groups —each group was constructed with seven mice.

**Figure 2:**
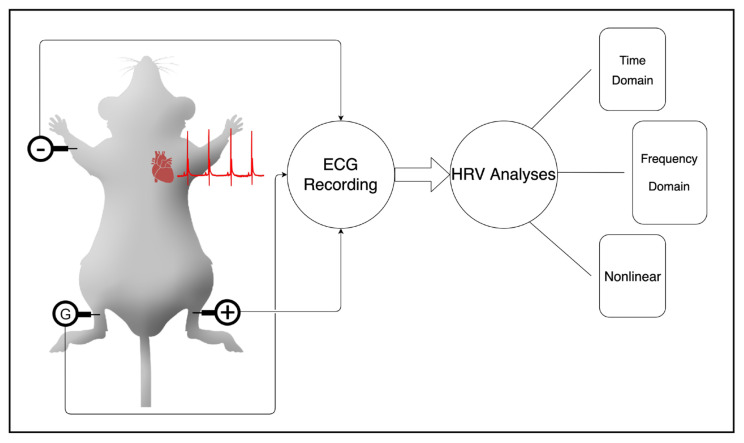
Representative ECG electrodes placement according to Lead II configuration and HRV analyses performed. (−): Negative electrode, (+): Positive electrode, (G): Ground electrode.

After the optimal anesthesia level was achieved, the mice were placed on a heated thermal plate in the supine position to obtain ECG recordings. For Lead II, the needle ECG electrodes were located under the skin of the right arm and the left leg (Figure 2) [21]. Surface ECG recordings were obtained using PowerLab/SP8 (ADInstruments, Australia) with an ECG sampling frequency of 4 kHz. No notch filter was required, and the high-pass and low-pass filter settings were 0.3 Hz and 1 kHz, respectively. For “R” wave detection LabChart 7 software (ADInstruments, Australia) was used.

### 2.3. Heart rate variability analysis

The tachogram of RR intervals was obtained after R waves were detected using the Pan-Tompkins real-time QRS recognition algorithm [22]. Berger interpolation was used to convert these RR tachograms to time series. The same data sets were used for all the analyses; i.e. the same time series was used for the frequency domain and time domain analyses, as well as for nonlinear analyses.

#### 2.4.1. Time domain analysis

##### SDNN

The interval between consecutive heartbeats of normal sinus beats (SDNN) was calculated in milliseconds. “Normal” denoted the absence of irregular beats, such as ectopic beats. For the SDNN analysis, the standard short-term recording time was five minutes. SDNN is influenced by both parasympathetic nervous system (PNS) activity and sympathetic nervous system (SNS) activity, which is strongly correlated with very low frequency (VLF), and low frequency (LF) band power, and also total power [23]. The measurement conditions influence this relationship. These bands contribute more to SDNN because they have more influence than the high frequency (HF) band [24].

##### RMSSD

The root mean square of successive differences between regular heartbeats (RMSSD) was obtained by calculating each successive time difference between heartbeats in ms. Then, the square root of the sum was obtained after squaring each of the values and summing the result. The standard recording time for RMSSD is five minutes [24].

The RMSSD is the primary time-domain indicator used to estimate the vagal alterations observed in HRV. It represents the beat-to-beat variation in HR (12). The RMSSD is the same as the nonlinear Poincaré plot standard deviation 1 (SD1) metric, which measures short-term HRV (37). RMSSD measurements over 24-h are highly correlated with the percentage of successive RR intervals that differ by more than 50 ms (pNN50) and HF power [25].

Although the RMSSD has a positive correlation with HF power [26], the impact of respiration rate on this index is unknown. Across many functions, the RMSSD is less affected by respiration than the respiratory sinus arrhythmia. The PNS has a greater effect on the RMSSD than the SDNN [24].

##### TINN

The TINN is the width of the baseline in a histogram of normal-to-normal (NN) intervals [27]. Contamination by only two objects within a five-minute segment can greatly distort its meaning, similar to SDNN and RMSSD [28].

#### 2.4.2. Frequency domain analysis

For the frequency domain analysis, the Kubios HRV Software (University of Eastern Finland) was used. Each recording period was exemplified by four minutes of R-R tachogram and resampled with 10 Hz to convert to time series.

The frequency bands of our frequency-domain analysis, which were selected as proposed by Thireau et al. (2008) [29], were as follows: VLF: 0.00–0.15 Hz; LF: 0.15–1.5 Hz; HF: 1.5–5 Hz. LF/HF ratios were also evaluated. The relative powers of frequency bands, namely the percentage of power spectrum densities (PSD%), were reported.

#### 2.4.3. Nonlinear analysis

##### Detrended fluctuation analysis (DFA)

RR time series were analyzed using Kubios HRV Software (University of Eastern, Finland) according to the DFA algorithm introduced by Peng et al. (1995) [30]. To further analyze the fluctuations in time series, this method employs the detrending technique. The correlation between the fluctuation function and the sample size is expressed in the scaling exponents obtained from this analysis. The self-similarity of the time series is expressed by this relationship between them. There are several steps to this method: to begin, the sum of the differences between the average RR (RR_a_) and each RR value (RR_i_) in the time series yields the integrated time series y(k) (Eq. 1).


Equation (1) $$y(k)=\sum_{i=1}^{k}\left[{RR}_{i}-{RR}_{a}\right]$$

The time series *y*(*k*) is divided into nonoverlapping (n) boxes of equal size. The local trend *y_n_*(*k*) for each box is determined for detrending integrated time series and then detrended by subtracting from the integrated time series *y*(*k*). The root-mean-square of the detrended time series obtained in the previous step is used to measure the fluctuation function *F*(*n*) (Eq. 2):


Equation (2) $$F(n)=\sqrt{\frac{1}{N}\sum_{k=1}^{N}{\left[y(k)-y_{n}(k)\right]}^{2}}$$

where F(n) is the fluctuation function of box size n, N is the number of the value in the time series, y(k) is the integrated time series, and *y_n_*(*k*) is the local trend series in each box.

To determine the correlation properties of the time series, the logarithmic relationship between the fluctuation function *F*(*n*) and the box size n is scaled. Calculating the slope of this logarithmic relation yields the scaling exponents DFA*α_1_* and DFA*α_2_*.

Short-term (DFA*α_1_*) and long-term (DFA*α_2_*) scaling exponents were determined in both groups for different box sizes 4 < n < 11 and 12 < n < 64, respectively [31].

##### Poincaré plot analysis

The Poincaré plot was analyzed by drawing an ellipse around the plotted points using the Kubios HRV Program (University of Eastern, Finland). From the analysis viewpoint, the Poincaré plot is a statistical visual analysis method. Regardless of the time series’ longitude, the correlation spectrogram obtained by the Poincaré plot allows for accurate recognition of the time series. Each pair of RR intervals (previous and next) are located in the rectangular coordinate system according to their coordinates (x, y), where x represents the RR_n_ interval value, and y represents the RR_n+1_ [32]. The graph’s dispersion generates a segment of points whose center is on the line known as the “line of identity”, which is the graph of the function x = y (RR_n_ = RR_n+1_). RR intervals longer than the previous one are represented by points above the line of identity, and intervals shorter than the previous one, represented by points below the line of identity [33]. These coordinates are used to measure the following three parameters: (i) SD1, (ii) SD2, and (iii) SD2/SD1, all of which were included in the present study.

- SD1, which determines the ellipse’s width, is the standard deviation of each point’s distance from the y = x axis. SD1 was considered to correlate with blood pressure changes, and power in the LF and HF bands, and total power of frequency domain analysis which is obtained from short-term recordings of five minutes [34].
- SD2, which determines the ellipse’s length, is the standard deviation of each point from the y = x + average R–R interval. SD2 is thought to be a reflection of LF band power and baroreflex sensitivity [35].
- SD2/SD1 ratio was assumed to be the analog of the LF/HF ratio [36].

##### Entropy analysis

The entropy analysis was carried out with the Kubios HRV Program (University of Eastern, Finland). Approximate entropy (ApEn) and sample entropy (SampEn) measure the probability in a given sequence of length N and runs of models that are close to m points remain close (less than a certain tolerance level r) to m + 1 points. There is no fail-safe law for selecting r and m parameters. Generally, values of the standard HRV deviation between 10% and 25% (most often, 20%) can be used. Commonly, m = 2 is used for model length, and m = 1 for very short time series [37].

Estimated entropy is used to determine the regularity and complexity of a time series. ApEn is optimized for a short time series with possible noise, and provides no details about the dynamics of the underlying mechanism. When applied to HRV data, large ApEn values indicate poor predictability of fluctuations in successive RR intervals. Small ApEn values suggest that the signal is normal and predictable [24].

To calculate sample entropy, a less biased and more accurate measurement of signal accuracy and complexity has been developed. The values of SampEn are converted to ApEn and can be measured using fewer than 200 values in a much shorter time series [24].

### 2.4. Statistical analysis

Firstly, the Shapiro–Wilk test was used to determine whether the data were normally distributed. Since this seemed to be the case, we then used a parametric statistical approach to further analyze our data. The statistical significance between the two groups was analyzed using the independent sample t-test, and for the multiple comparisons, one-way ANOVA was used. The IBM SPSS Statistics Version 21.0 (IBM Corporation, Armonk, NY, USA) was used for statistical analyses, and the statistical significance level was adjusted to p < 0.05. The data were expressed as ‘mean ± SD (standard deviation)’.

## 3. Results

### 3.1. Time domain analysis

**Figure 3:**
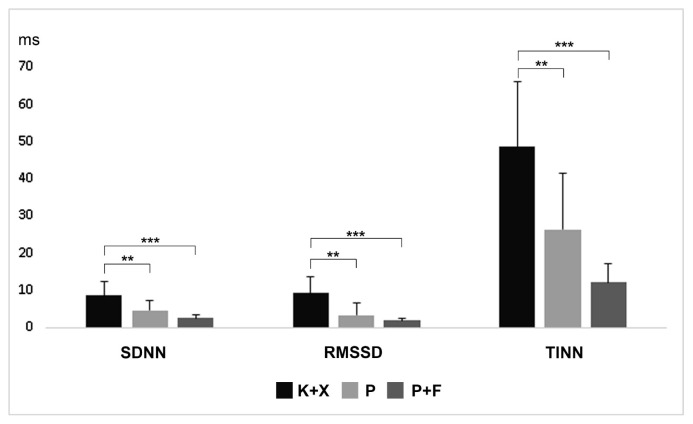
Effects of ketamine+xlazine (K+X), pentobarbital (P), and pentobarbital+fentanyl (P+F) on time-domain parameters of HRV analysis.

The time-domain analysis results were compared as shown in Figure 3: SDNN was calculated as 8.57 ± 3.69 ms in group K+X, 4.58 ± 2.78 ms in group P, and 2.498 ± 1.00 ms in group P+N. RMSSD was measured as 9.27 ± 4.20 ms in group K+X, 3.34 ± 3.19 ms in group P, and 1.87 ± 0.58 ms in group P+N. TINN was calculated as 48.57 ± 17.72 ms in group K+X, 26.42 ± 15.19 ms in group P, and 12.14 ± 4.88 ms in group P+N. SDNN, RMSSD, and TINN were reduced significantly in group P (p < 0.01) and P+F (p < 0.001) compared to Group K+X. SDNN, RMSSD, and TINN did not differ significantly between group P and group P+F (p > 0.05).

**Figure 4:**
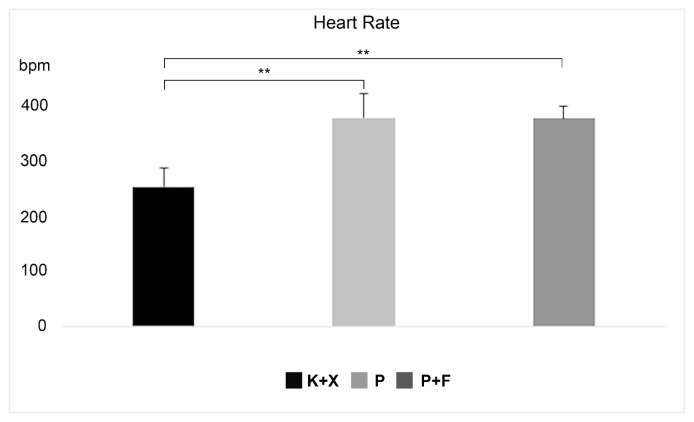
Effects of ketamine+xlazine (K+X), pentobarbital (P), and pentobarbital+fentanyl (P+F) on the HR.

Mean HR was 253.81 ± 34.61 bpm, 376.39 ± 23.62 bpm and 377.99 ± 42.94 bpm in group K+X, group P, and group P+F, respectively. Note that the mean HR was significantly lower in group K+X in comparison to the others (p < 0.01) but did not differ significantly between group P and group P+F (p > 0.05) (Figure 4).

### 3.2. Frequency domain analysis

**Figure 5:**
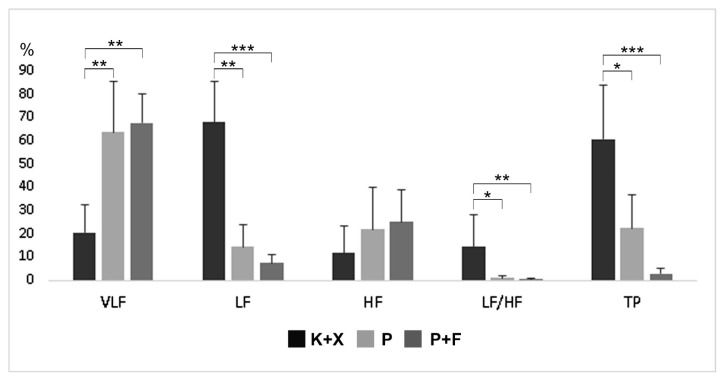
Effects of ketamine+xlazine (K+X), pentobarbital (P), and pentobarbital+fentanyl (P+F) on frequency-domain parameters of HRV analysis.

The mean relative VLF power was 63.69 ± 21.84, 67.61 ± 12.54, and 20.43 ± 12.05 in group K+X, group P, and group P+F, respectively. Relative VLF power was significantly lower in group K+X compared to group P and group P+F (p < 0.01). Relative VLF power did not differ significantly between group P and group P+F (p > 0.05) (Figure 5). The mean relative LF power was 14.34 ± 9.65, 7.3 ± 3.74, and 67.96 ± 17.05 in group K+X, group P, and group P+F, respectively. Relative LF power was significantly higher in group K+X compared to group P (p < 0.01) and group P+F (p < 0.001) (Figure 5). Relative LF power did not differ significantly between group P and group P+F (p > 0.05). The mean relative HF power was 21.96 ± 17.85, 25.09 ± 13.54, and 11.60 ± 11.50 in group K+X, group P, and group P+F, respectively. The changes in relative HF power were not statistically significant between groups (p > 0.05). The LF/HF ratio was 14.47 ± 13.69, 1.03 ± 1.00, and 0.48 ± 0.43 in group K+X, group P, and group P+F, respectively. The LF/HF ratio was significantly higher in group K+X compared to group P (p < 0.05) and group P+F (p < 0.01) but did not significantly differ between group P and group P+F (p > 0.05) (Figure 5). Total power (TP) was 22.42 ± 14.18, 2.86 ± 2.41, and 60.71 ± 23.074 in group K+X, group P, and group P+F, respectively (Figure 5). TP was significantly higher in group K+X compared to group P (p < 0.05) and group P+F (p < 0.001). TP did not differ significantly between group P and group P+F (p > 0.05) (Figure 5).

### 3.3. Detrended fluctuation analysis (DFA)

**Figure 6:**
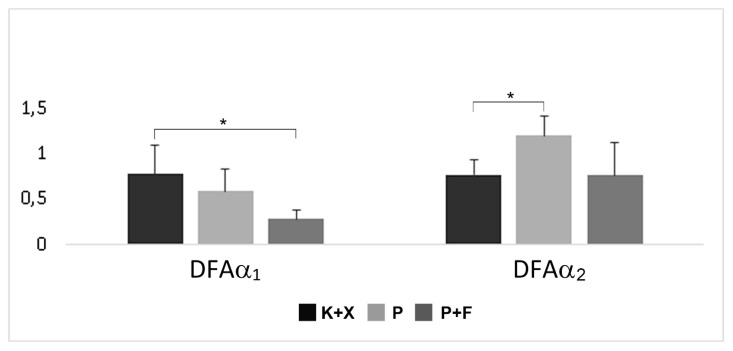
Effects of ketamine+xlazine (K+X), pentobarbital (P), and pentobarbital+fentanyl (P+F) on detrended fluctuation analysis.

Detrended fluctuation analysis results were compared between the groups. Mean DFAα_1_ was calculated as 0.28 ± 0.09, 0.77 ± 0.31 and 0.58 ± 0.25 in group K+X, group P, and group P+F, respectively. Mean DFAα_2_ was calculated as 1.19 ± 0.21, 0.76 ± 0.16 and 0.76 ± 0.36 in group K+X, group P, and group P+F, respectively. DFAα_1_ was higher in group K+X compared to group P+F (p < 0.05). DFAα_1_ changes between group P and group P+F, and between group K+X and group P were not statistically significant (p > 0.05) (Figure 6). DFAα_2_ was lower in group K+X compared to group P (p < 0.05). DFAα_2_ changes between group P and group P+F, and between group K+X and group P+F were not statistically significant (p > 0.05) (Figure 6).

### 3.4. Poincaré plot analysis

**Figure 7:**
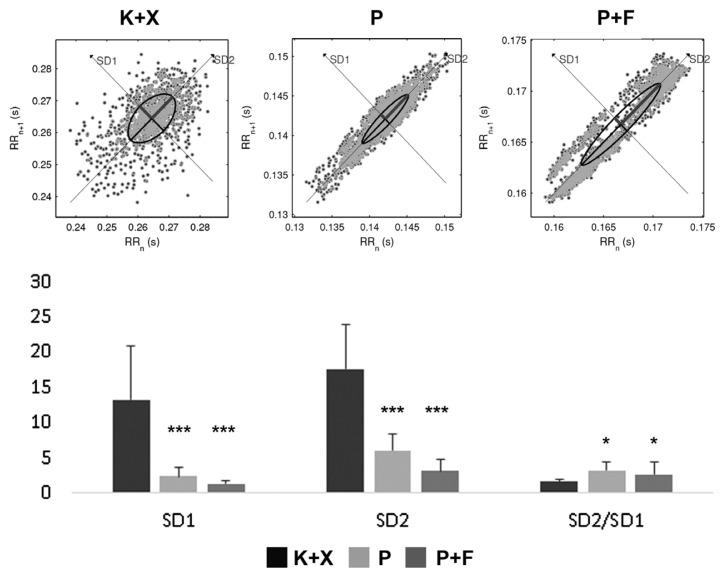
Effects of ketamine+xlazine (K+X), pentobarbital (P), and pentobarbital+fentanyl (P+F) on Poincaré plot analysis.

Poincaré plot parameters SD1, SD2, and SD2/SD1 ratio were compared between the groups. SD1 was 13.28 ± 7.60 ms, 2.34 ± 1.25 ms, and 1.34 ± 0.42 ms in group K+X, group P, and group P+F, respectively. SD2 was 17.59 ± 6.23 ms, 3.14 ± 1.60 ms, and 2.63 ± 1.82 ms in group K+X, group P, and group P+F, respectively. The SD2/SD1 ratio was 1.57 ± 0.39, 3.19 ± 1.27, and 2.63 ± 1.82 in group K+X, group P, and group P+F, respectively. Both SD1 and SD2 were significantly higher in group K+X compared to group P and group P+F (p < 0.001) but did not differ significantly between group P and group P+F (p > 0.05). The SD2/SD1 ratio was significantly lower in group K+X compared to group P and group P+F (p < 0.05), however, did not differ between group P and group P+F (p > 0.05) (Figure 7).

### 3.5. Entropy analysis

**Figure 8:**
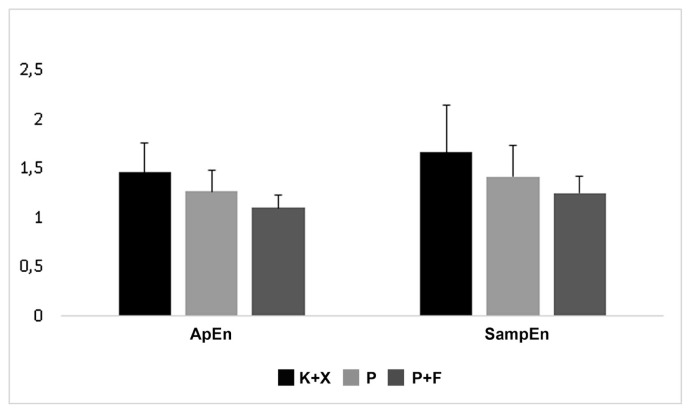
Effects of ketamine+xlazine (K+X), pentobarbital (P), and pentobarbital+fentanyl (P+F) on entropy analysis.

In the entropy analysis, ApEn and SampEn were compared between the groups (Figure 8). The calculated mean ApEn values were 1.46 ± 0.29, 1.26 ± 0.22 and 1.10 ± 0.13 in group K+X, group P, and group P+F, respectively. The calculated mean SampEn values were 1.66 ± 0.48, 1.42 ± 0.31, 1.25 ± 0.17 in group K+X, group P, and group P+F, respectively. Both mean ApEn and SampEn values did not differ significantly between groups (p > 0.05) (Figure 8).

## 4. Discussion

The major findings of this study may be summarized as follows: (i) the pentobarbital (in group P) and pentobarbital fentanyl (in group P+F), caused a more stable HR during general anesthesia than the kethamine+xlazine (in group K+X) combination; (ii) the greatest reduction in complexity parameters during general anesthesia was seen in the group P+N; (iii) the irregularity-reflecting parameters SampEn and ApEn did not differ between groups; (iv) time-domain parameters, SDNN, RMSSD and TINN, behaved in an opposite manner to the frequency domain and nonlinear parameters.

In cardiac research, importance is attached to the selection of anesthetic agents, because these vary in their effects on hemodynamics, and can increase the incidence of arrhythmias. In terms of cardiovascular study, no single ideal anesthetic agent has been identified, and the use of a combination of drugs is appropriate because it allows for the benefits of small doses without the drawbacks of large doses of any one medication [38]. Using large doses of these anesthetics, especially pentobarbital, causes cardiac depression [5].

The results of our study showed that the K+X combination for anesthesia created more stable cardiovascular conditions than either the P+F combination or pentobarbital. Pentobarbital has been shown to induce hypotension and tachycardia [39], but in lower concentrations (approximately 30 mgkg^−1^) it has been shown not have a depressive effect on the heart [14, 39]. Under pentobarbital anesthesia in mice, Xiuying et al. (2002) and Yan et al. (2009) found persistence in activity of autonomic nerves, including vagal efferents [40,41]. In the present study, using a high dose of pentobarbital (90 mgkg^−1^) decreased complexity, which may have been caused by the drug’s antivagal effect [5].

The effects of pentobarbital on myocardial function have been studied in dogs [42] and pigs [43]. Some authors have reported the depressive effects of pentobarbital on the myocardium [42,43]. The systemic vascular system is also considered to be impaired by pentobarbital anesthesia. Pentobarbital inhibits synaptic transmission in sympathetic ganglia and catecholamine release from the adrenal medulla, as well as depressing the carotid baroreceptor reflex function [4].

In our study, the bradycardia effect in the K+X combination group was more severe than in the other groups. However, during the general anesthesia periods, the mean DFAα_1_ in K+X anesthesia was closer to 1, compared to group P and group P+F. Peng et al. (1995) stated in the article that first introduced the DFA algorithm that the DFAα exponent (box size: 4–16), which corresponds to DFAα_1_ in our study, is likely due to the physiological interbeat interval fluctuation that is dominated by the relatively smooth heartbeat oscillation associated with respiration on very short time scales [30]. Xylazine can cause severe cardiopulmonary depression. Respiratory rate and HR decrease after the administration of Xylazine [44]. Following xylazine administration, the respiratory rate and HR were found to be decreased [11]. Our findings of lower HR and HF power in the group K+X combination were in agreement with these findings [11, 44]. DFAα_1_was higher in group K+X, suggesting that, as expected, the combination of K+X did not affect respiratory-related complexity parameters.

Another combination examined in the present study was the P+F combination. Fentanyl alone, according to Flacke et al., can cause hypotension by reducing sympathetic outflow [45]. They demonstrated the absence of hemodynamic effects of fentanyl in dogs deprived of the autonomic tone, suggesting that its effects are entirely mediated by the ANS. The results of the present study reveal that the addition of fentanyl to pentobarbital decreased the frequency-domain parameters compared to the sole administration of pentobarbital (Figure 5). Nevertheless, there was no difference in complexity and regularity measures between the group P and the group P+F (Figures 6–8). With the P+F combination, the required dose of pentobarbital can be reduced, and, therefore, more stable hemodynamic parameters can be obtained during general anesthesia. In the present study, however, we deliberately avoided lowering the dose of pentobarbital in the P+F combination group to evaluate the combined effects. Compared to group P and group P+F, the K+X combination reduced HR more severely; nevertheless, this combination had minimal effects on linear and nonlinear parameters of HRV analysis. Thus, it can be concluded that the K+X combination may preserve cardiovascular function better compared to the sole application of pentobarbital and the P+F combination. A possible explanation is that the K+X combination inhibits adrenergic components without affecting vagal components, and that this inhibition is not sufficiently severe to change or alter HRV parameters.

Because only a single dose of the drugs was administered, our study provides no evidence on the effect on ANS of the anesthetics at increasing doses and our results can only suggest the effects of anesthetics in higher doses. Further studies are needed that evaluate dose-dependent effects of anesthetics on ANS.

Before initiation of the study protocol, the depth of anesthesia was evaluated by the pedal withdrawal reflex as well as by the breathing frequency (2.1. Animals and anesthesia). When the animal remains calm and quiet, is insensitive to external stimuli, and has steady heart and breathing rates at around 60, the depth of anesthesia is considered to be optimal [3]. Although the absence of the palpebral reflex in mice indicates a good anesthetic depth, the pedal withdrawal reflex cannot be used to provide quantitative data on the depth of anesthesia, which requires relatively new depth-of-anesthesia evaluation methods, such as epinephrine or norepinephrine measurements and bispectral index. A limitation of the current study that such methods were not employed. However, it should be noted that the HR itself is more sensitive than arterial blood pressure as a parameter for evaluating the anesthesia level [46]. For decades, in addition to HR measurements, several HRV analyses were performed to assess the effects of anesthesia on ANS [5–7, 45].

Also, a general limitation must be addressed. The value of HRV interpretation has often been questioned because HRV represents HR regulation by the parasympathetic and sympathetic nervous systems, rather than absolute levels of autonomic activity [47]. Recent studies have shown that HRV is a useful tool for assessing the impact of autonomic oscillations on heart rate, especially during anesthesia [48]. However, since certain physiological reflexes or nonneural mechanisms may be inadequately reflected in the HRV signal, some autonomic control mechanisms may not be fully explained by this study [49].

Animal experimentation is a long-standing practice in medicine, especially for testing new therapies in animal models before translating their findings to humans [46]. However, not all results achieved on animals can be directly translated to human, and of the many possible reasons for this, one may be the lack of data on the effects of anesthetics. We believe that, by shedding light on the effects of the most commonly used anesthetics in animal studies, our data will contribute to the literature on the translation of the results from animal studies into medical practices.

Anesthetic side effects can affect the accuracy of experimental results and should be avoided as far as possible. The depressed cardiac variability caused by the pentobarbital combination means that this anesthetic should be excluded from the studies that focus on evaluating the ANS functions. K+X has fewer hemodynamic effects in rats compared to pentobarbital or the pentobarbital+fentanyl combination. When all our findings are considered together, it appears that the K+X combination caused more extensive bradycardia, yet this combination is the most effective anesthetic agent for evaluating cardiac autonomic effects interventions under the conditions of this study.

## 5. Conclusion

In conclusion, our study demonstrated that K+X administration in mice leads to sympathetic withdrawal, with a trend toward vagal activation; without, however, severely affecting ANS modulation. HRV analyses, including nonlinear methods, indicated that the K+X combination produces lower levels of imbalance and disorder in the regulation of the ANS, in comparison to both pentobarbital and the P+F combination. In the studies focusing on the effects on ANS, K+X anesthesia may be preferred to pentobarbital and the P+F combination.

## References

[1] McCraty R Shaffer F Heart rate variability: new perspectives on physiological mechanisms, assessment of self-regulatory capacity, and health risk Global Advances in Health And Medicine 2015 4 1 46 66 10.7453/gahmj.2014.073 PMC4311559 25694852

[2] Uchino BN Uno D Holt-Lunstad J Flinders JB Age-related differences in cardiovascular reactivity during acute psychological stress in men and women The Journals of Gerontology Series B: Psychological Sciences and Social Sciences 1999 54 6 339 346 10.1093/geronb/54b.6.p339 10625961

[3] Gargiulo S Greco A Gramanzini M Esposito S Affuso A Brunetti A Mice anesthesia, analgesia, and care, Part I: anesthetic considerations in preclinical research Institute for Laboratory Animal Research 2012 53 1 55 69 10.1093/ilar.53.1.55 23382271

[4] Goldmann C Ghofrani A Hafemann B Fuchs P Khorram-Seffat R Afify M Combination anesthesia with ketamine and pentobarbital: a long-term porcine model Research in Experimental Medicine 1999 199 1 35 50 10494673 10.1007/s004330050131

[5] Chiba S Tsuboi M Dominant anti-vagal effect of pentobarbital on cardiac responses to intracardiac autonomic nerve stimulation in the dog Japanese Journal of Pharmacology 2001 86 2 248 250 10.1254/jjp.86.248 11459129

[6] Johnstone M The cardiovascular effects of ketamine in man Anaesthesia 1976 31 7 873 882 10.1111/j.1365-2044.1976.tb11898.x 970587

[7] Kuo TB Lai CJ Huang YT Yang CC Regression analysis between heart rate variability and baroreflex-related vagus nerve activity in rats Journal of Cardiovascular Electrophysiology 2005 16 8 864 869 10.1111/j.1540-8167.2005.40656.x 16101628

[8] Bauer JA Fung HL Concurrent hydralazine administration prevents nitroglycerin-induced hemodynamic tolerance in experimental heart failure Circulation 1991 84 1 35 39 10.1161/01.cir.84.1.35 1905595

[9] Baum D Halter JB Taborsky GJ Jr Porte D Jr Pentobarbital effects on plasma catecholamines: temperature, heart rate, and blood pressure American Journal of Physiology 1985 248 1 95 100 10.1152/ajpendo.1985.248.1.E95 3966554

[10] Hosomi H Sagawa K Effect of pentobarbital anesthesia on hypotension after 10% hemorrhage in the dog American Journal of Physiology 1979 236 4 607 612 10.1152/ajpheart.1979.236.4.H607 434228

[11] Shekarforoush S Fatahi Z Safari F The effects of pentobarbital, ketamine-pentobarbital and ketamine-xylazine anesthesia in a rat myocardial ischemic reperfusion injury model Lab Anim 2016 50 3 179 184 10.1177/0023677215597136 26224732

[12] Watkins L Maixner W The effect of pentobarbital anesthesia on the autonomic nervous system control of heart rate during baroreceptor activation Journal of Autonomic Nervous System 1991 36 2 107 114 10.1016/0165-1838(91)90106-d 1765616

[13] Moore KA Ripple MG Sakinedzad S Levine B Fowler DR Tissue distribution of xylazine in a suicide by hanging Journal of Analytical Toxicology 2003 27 2 110 112 10.1093/jat/27.2.110 12670006

[14] Jiang X Gao L Zhang Y Wang G Liu Y Yan C Comparison of the effects of ketamine, chloral hydrate and pentobarbital sodium anesthesia on isolated rat hearts and cardiomyocytes Journal of Cardiovascular Medicine 2011 12 10 732 735 10.2459/JCM.0b013e32834a6697 21873882

[15] Liu D Shao Y Luan X Zhang M Shui C Wu Q Comparison of Ketamine-Pentobarbital anesthesia and Fentanyl-Pentobarbital anesthesia for open-heart surgery in minipigs Lab Animal 2009 38 7 234 340 10.1038/laban0709-234 19543261

[16] Becker LD Paulson BA Miller RD Severinghaus JW Eger EI 2nd Biphasic respiratory depression after fentanyldroperidol or fentanyl alone used to supplement nitrous oxide anesthesia Anesthesiology 1976 44 4 291 296 10.1097/00000542-197604000-00003 1259186

[17] Murphy MR Olson WA Hug CC Jr Pharmacokinetics of 3H-fentanyl in the dog anesthetized with enflurane Anesthesiology 1979 50 1 13 19 10.1097/00000542-197901000-00004 760597

[18] Hug CC Jr Murphy MR Fentanyl disposition in cerebrospinal fluid and plasma and its relationship to ventilatory depression in the dog Anesthesiology 1979 50 4 342 349 10.1097/00000542-197904000-00011 434538

[19] Sato N Miyake S Akatsu J Kumashiro M Power spectral analysis of heart rate variability in healthy young women during the normal menstrual cycle Psychosomatic Medicine 1995 57 4 331 335 10.1097/00006842-199507000-00004 7480562

[20] Gaertner DJ Hallman TM Hankenson FC Batchelder MA Anesthesia and Analgesia for Laboratory Rodents Fish RE Brown MJ Danneman PJ Karas AZ American College of Laboratory Animal Medicine, Anesthesia and Analgesia in Laboratory Animals 2nd Ed Academic Press 2008 239 297 10.1016/B978-012373898-1.50014-0

[21] Ha TW Oh B Kang JO Electrocardiogram recordings in anesthetized mice using Lead II Journal of Visualized Experiments 2020 160 10.3791/61583 32628173

[22] Singh S Pattern analysis of different ECG signal using Pan-Tompkin’s algorithm 1st International Journal on Computer Science and Engineering 2010 2 7 2502 2505

[23] Umetani K Singer DH McCraty R Atkinson M Twenty-four hour time domain heart rate variability and heart rate: relations to age and gender over nine decades Journal of the American College of Cardiology 1998 31 3 593 601 10.1016/s0735-1097(97)00554-8 9502641

[24] Shaffer F Ginsberg JP An overview of heart rate variability metrics and norms Front Public Health 2017 5 258 10.3389/fpubh.2017.00258 PMC5624990 29034226

[25] Bigger JT Jr Albrecht P Steinman RC Rolnitzky LM Fleiss JL Cohen RJ Comparison of time- and frequency domain-based measures of cardiac parasympathetic activity in Holter recordings after myocardial infarction American Journal of Cardiology 1989 64 8 536 538 10.1016/0002-9149(89)90436-0 2773799

[26] Huikuri HV Mäkikallio TH Perkiömäki J Measurement of heart rate variability by methods based on nonlinear dynamics Journal of Electrocardiology 2003 36 95 99 10.1016/j.jelectrocard.2003.09.021 14716599

[27] Kleiger RE Stein PK Bigger JT Jr Heart rate variability: measurement and clinical utility Annals of Noninvasive Electrocardiology 2005 10 1 88 101 10.1111/j.1542-474X.2005.10101.x 15649244 PMC6932537

[28] Tarvainen MP Niskanen JP Lipponen JA Ranta-Aho PO Karjalainen PA Kubios HRV-heart rate variability analysis software Computer Methods and Programs in Biomedicine 2014 113 1 210 220 10.1016/j.cmpb.2013.07.024 24054542

[29] Thireau J Zhang BL Poisson D Babuty D Heart rate variability in mice: a theoretical and practical guide Experimental Physiology 2008 93 1 83 94 10.1113/expphysiol.2007.040733 17911354

[30] Peng CK Havlin S Stanley HE Goldberger AL Quantification of scaling exponents and crossover phenomena in nonstationary heartbeat time series Chaos 1995 5 1 82 87 10.1063/1.166141 11538314

[31] Lin TT Sung YL Wu CE Zhang H Liu YB Lin SF Proarrhythmic risk and determinants of cardiac autonomic dysfunction in collagen-induced arthritis rats BMC BMC Musculoskeletal Disorders 2016 17 1 491 10.1186/s12891-016-1347-6 27894284 PMC5127040

[32] Tayel NB AlSaba EI Poincaré plot for heart rate variability International Journal of Biomedical and Biological Engineering 2015 9 9 708 711 10.5281/zenodo.1109321

[33] Roy S Goswami DP Sengupta A Geometry of the Poincaré plot can segregate the two arms of autonomic nervous system - A hypothesis Medical Hypotheses 2020 138 109574 10.1016/j.mehy.2020.109574 32014816

[34] Zerr CL Bartochowski Z Shrestha R Moessner A Shaffer F Does Inhalation-to-Exhalation Ratio Matter in Heart Rate Variability Biofeedback? Proceedings of the 28th Annual Student Research Conference Kirksville, MO, USA 10.1007/s10484-015-9282-0

[35] Brennan M Palaniswami M Kamen P Poincaré plot interpretation using a physiological model of HRV based on a network of oscillators American Journal of Physiology-Heart and Circulatory 2002 283 5 1873 1886 10.1152/ajpheart.00405.2000 12384465

[36] Motie-Nasrabadi A Behbahani S Dabanloo NJ Ictal heart rate variability assessment with focus on secondary generalized and complex partial epileptic seizures Advances in Bioresearch 2013 4 1 50 58

[37] Sassi R Cerutti S Lombardi F Malik M Huikuri HV Peng CK Schmidt G Yamamoto Y Advances in heart rate variability signal analysis: joint position statement by the e-Cardiology ESC Working Group and the European Heart Rhythm Association co-endorsed by the Asia Pacific Heart Rhythm Society Europace 2015 17 9 1341 1353 10.1093/europace/euv015 26177817

[38] Grund F Tjomsland O Sjaastad I Ilebekk A Kirkebøen KA Pentobarbital versus medetomidine-ketamine-fentanyl anaesthesia: effects on haemodynamics and the incidence of ischaemia-induced ventricular fibrillation in swine Lab Animal 2004 38 1 70 78 10.1258/00236770460734425 14979991

[39] Murthy VS Zagar ME Vollmer RR Schmidt DH Pentobarbital-induced changes in vagal tone and reflex vagal activity in rabbits European Journal of Pharmacology 1982 84 1–2 41 50 10.1016/0014-2999(82)90155-8 7140820

[40] Ma X Abboud FM Chapleau MW Analysis of afferent, central, and efferent components of the baroreceptor reflex in mice American Journal of Physiology Regulatory, Integrative and Comparative Physiology 2002 283 5 1033 1140 10.1152/ajpregu.00768.2001 12376395

[41] Yan B Li L Harden SW Epstein PN Wurster RD Cheng ZJ Diabetes induces neural degeneration in nucleus ambiguus (NA) and attenuates heart rate control in OVE26 mice Experimental Neurology 2009 220 1 34 43 10.1016/j.expneurol.2009.07.006 19615367

[42] Nash CB Floyd D Woodbury RA Cardiovascular effects of anesthetic doses of pentobarbital sodium American Journal of Physiology-Legacy Content 1956 185 1 107 112 10.1152/ajplegacy.1956.185.1.107 13313756

[43] Sawyer DC Lumb WV Stone HL Cardiovascular effects of halothane, methoxyflurane, pentobarbital, and thiamylal Journal of Applied Physiology 1971 30 1 36 43 10.1152/jappl.1971.30.1.36 5538792

[44] Atalan G Demirkan I Güneş V Cihan M Celebi F Citil M Comparison of xylazine+ ketamine-HCI anaesthetic agents with acepromazine+ butorphanol+ ketamine combinations for their clinical and cardiorespiratory effects in dogs Veteriner Cerrahi Dergisi 2002 8 3–4 35 40

[45] Flacke JW Davis LJ Flacke WE Bloor BC Van Etten AP Effects of fentanyl and diazepam in dogs deprived of autonomic tone Anesthesia & Analgesia 1985 64 11 1053 1059 4051203

[46] Ozbek M Domack U Barnikol WK Eine erweiterte Evaluation der Neurolept-Anästhesie für Meerschweinchen mit einer Analyse gemischt-exspiratorischer Gase während Spontanatmung. Wirkung des Fastens auf das kardiorespiratorische System und den Metabolismus An extended evaluation of a neuroleptanesthesia for the guinea pig with analysis of mixed expiratory gases during spontaneous breathing. Effects of fasting on the cardiorespiratory system and metabolism. (Article in German with an abstract in English) Journal of Animal Physiology and Animal Nutrition 2004 88 1–2 20 29 10.1111/j.1439-0396.2004.00450.x 19774759

[47] Haney MF Wiklund U Can heart rate variability become a screening tool for anesthesia-related hypotension? Acta Anaesthesiologica Scandinavica 2007 51 10 1289 1291 10.1111/j.1399-6576.2007.01517.x 17944628

[48] Vettorello M Colombo R De Grandis CE Costantini E Raimondi F Effect of fentanyl on heart rate variability during spontaneous and paced breathing in healthy volunteers Acta Anaesthesiologica Scandinavica 2008 52 8 1064 1070 10.1111/j.1399-6576.2008.01713.x 18840105

[49] Parati G Mancia M Di Rienzo M Castiglioni P Taylor JA Cardiovascular variability is/is not an index of autonomic control of circulation Journal of Applied Physiology 2006 101 2 690 691 10.1152/japplphysiol.00584.2006 16645191

